# Inhibiting melanoma tumor growth: the role of oxidative stress-associated *LINC02132* and *COPDA1* long non-coding RNAs

**DOI:** 10.3389/fimmu.2025.1558292

**Published:** 2025-02-28

**Authors:** JingWen Xu, MingZhu Jin, ZhenZhen Mu, ZhengXiu Li, RuiQun Qi, XiuPing Han, HangHang Jiang

**Affiliations:** ^1^ Department of Dermatology, Shengjing Hospital of China Medical University, Shenyang, China; ^2^ Department of Dermatology & Key Lab of Dermatology, Ministry of Education and Public Health, National Joint Engineering Research Center for Theranostics of Immunology Skin Diseases, The First Hospital of China Medical University, Shenyang, China

**Keywords:** melanoma, lncRNA, oxidative stress, prognosis, *LINC02132*, *COPDA1*

## Abstract

**Background:**

Cutaneous melanoma is a type of malignant tumor that is challenging to predict and is readily stimulated by various factors. Oxidative stress can induce damage and alterations in melanocytes, subsequently triggering immune responses. Given that oxidative stress is a prevalent tumor stimulus, we aimed to enhance melanoma prediction by identifying lncRNA signatures associated with oxidative stress.

**Methods:**

We screened for oxidative stress-related lncRNAs that could improve melanoma patient prognosis using the TCGA and GTEx databases. Utilizing differentially expressed oxidative stress-related lncRNAs (DE-OSlncRNAs), we constructed a Lasso regression model. The accuracy of the model was validated using univariate and multivariate regression, Kaplan-Meier (K-M) curves, and ROC curves. Subsequently, we conducted immune infiltration analysis, immune checkpoint differential analysis, IC50 pharmaceutical analysis, and gene set enrichment analysis. Investigating the effects of the target gene on melanoma using fluorescence in situ hybridization (FISH), quantitative real-time PCR (qRT-PCR), Edu assay, wound healing assay, transwell assay, flow cytometry, and reactive oxygen species (ROS) detection.

**Results:**

Thirteen lncRNAs were identified as significant prognostic factors. Four oxidative stress-related lncRNAs (*COPDA1*, *LINC02132*, *LINC02812*, and *MIR205HG*) were further validated by fluorescence in situ hybridization (FISH), with results consistent with our data analysis. *LINC02132* and *COPDA1* can influence the proliferation, invasion, migration, and apoptosis of melanoma.

**Conclusion:**

Our findings suggest that upregulation of the *LINC02132* or *COPDA1* genes elevates intracellular reactive oxygen species (ROS) levels in melanoma cells, suppresses tumor cell proliferation, migration, and invasion, and promotes apoptosis. These results suggest a novel therapeutic strategy for melanoma treatment.

## 1 Introduction

Cutaneous melanoma is a malignant cancer characterized by a high incidence rate and fatality ratio (1). The prognosis of melanoma is typically influenced by environmental exposure, genetic factors, and the surrounding immune microenvironment (2). Following the development of immune checkpoint blockade and targeted molecular pharmaceuticals, traditional treatments such as chemotherapy or radiation therapy have been gradually replaced due to their extensive damage (3). However, every approach has its drawbacks. Immunotherapy may offer a promising start for treating metastatic melanoma, yet as treatment progresses, issues such as intrinsic resistance and diminishing therapeutic effects arise (4). Consequently, minimizing time and financial burdens for patients while providing precise, individualized treatments becomes a priority in melanoma therapy. Oxidative stress is a ubiquitous factor in all organic life; it may serve as a defense mechanism to eliminate pathogens (5) and also as a barrier to restrict the migration and metastasis of melanoma cells (6). Antioxidant extracts (7) and doxycycline (8) have been shown to promote apoptosis and cytotoxicity in melanoma cell lines. This effect is contingent upon the highly elevated oxidation levels in melanoma (9); when this balance is disrupted, cells may activate antioxidant signaling pathways to counteract environmental exposure (10). Similarly to microbes, melanoma cells develop various capabilities to utilize ions in regulating their cellular functions, such as ferroptosis induced by reactive oxygen species (11), or restoring ROS accumulation through calcium channel activation in melanoma (12). Ultraviolet (UV) light is a significant risk factor for the development of melanoma. Increased melanin synthesis can protect cells from UV-induced damage. However, the process of melanin synthesis also generates reactive oxygen species (ROS). Low levels of ROS can promote the transition from the G1 to the S phase of the cell cycle, thereby enhancing cell proliferation. Conversely, high levels of ROS can induce oxidative stress, leading to cell apoptosis (13, 14). Ultimately, any alteration in ion transport results in a disturbance of the oxidative state. To preserve normal cellular function, long non-coding RNAs (lncRNAs) regulate the cell cycle, cell migration, and epigenetic expression (15). Identifying specific lncRNAs offers significant potential in cancer therapy (16). Numerous studies have confirmed the involvement of specific lncRNAs in the regulation of various cancers. For instance, Jin W et al. discovered that lncRNA ZNRD1-AS1 promotes malignant lung cell proliferation, migration, and angiogenesis through the miR-942/TNS1 axis (17). Pengyu H et al. identified several immunotherapy-related lncRNAs that can predict bladder cancer and provide new insights for clinical drug development (18). Additionally, Jinlan G et al. revealed that *MIR205HG* supports melanoma growth via the miR-299-3p/VEGFA axis, positioning *MIR205HG* as a potential therapeutic target for melanoma treatment (19). Growth arrest-specific transcript 5 is a lncRNA that induces melanoma oxidation and apoptosis through the activation of CDKN1 (20). Consequently, this study integrates oxidative stress and the immune model to concentrate on predictive signatures, which will be validated using various databases. Utilizing this signature, patients may benefit from micro-molecule antioxidants, and it may also serve as a reference for timely therapeutic adjustments.

## 2 Materials and methods

### 2.1 Data introduction

Clinical and RNA sequencing data for skin cutaneous melanoma (TCGA-SKCM) were obtained from the Genomic Data Commons Data Portal (GDC, https://portal.gdc.cancer.gov/). Additionally, normal skin sequencing lncRNA data were downloaded from the Genotype-Tissue Expression (GTEx, https://gtexportal.org/home/) to serve as a control group.

### 2.2 Data preprocessing

Potential confounding factors were mitigated by standardizing the data using the voom function from the limma package (21) and log2 counts-per-million, followed by further normalization of the CPM values in the edgeR package (22). After standardizing the dataset, we performed batch effect correction using the ComBat method (23), resulting in a cleaned TCGA-SKCM dataset comprising 472 samples with 17,622 gene expression profiles.

### 2.3 Differential expression analysis

Differential expression analysis was performed using the edgeR package (22) to identify genes exhibiting differential expression under specific conditions. Specifically, genes related to metabolism were screened for differential expression, with the threshold set as |log2FC| > 1 and q-value < 0.05.

### 2.4 Correlation analysis

The correlation between all lncRNAs and oxidative stress-related genes was calculated using the psych package to identify oxidative stress-related lncRNAs. Genes with an absolute correlation value greater than 0.6 and a P-value less than 0.05 were considered significant. This process facilitated the identification of differentially expressed lncRNAs.

### 2.5 Construction of risk model

Utilizing differentially expressed lncRNAs related to oxidative stress genes and corresponding clinical data, univariate Cox regression analysis was conducted to screen for lncRNAs with P<0.05. A Lasso regression model was then constructed using gene expression values and corresponding clinical data from the TCGA database, employing the R package “glmnet” (24). Sample predictions were made based on their corresponding risk values as determined by this risk model.

### 2.6 Survival prognostic analysis

Cox regression analysis was performed using the survival package (25) on effective gene expressions obtained from Lasso regression (with nonzero regression coefficients) in cancer samples, in conjunction with clinical data. ROC analysis, based on risk values, was followed by COX regression analysis to assess risk values in clinical classifications. High-risk groups were defined as those exceeding the median risk score of their respective group, while low-risk groups fell below this threshold; survival analyses were conducted to verify these classifications.

### 2.7 Gene set enrichment analysis

Gene Set Enrichment Analysis (GSEA) (26) was employed to investigate the significant differences in gene sets based on gene expression conditions and data from the GO (27) and KEGG (28) databases. GSEA is a computational method used to assess whether a given gene set exhibits notable variations between different groups.

### 2.8 Immune infiltration analysis (CIBERSORT)

CIBERSORT (29) was initially utilized for immune infiltration analysis, using standardized cancer expression values obtained from TCGA. The differential statuses of immune cells between high and low-expression groups were then compared using the Wilcoxon rank-sum test.

### 2.9 Analysis of immune checkpoint relation

The Wilcox. Test function was applied to calculate and visualize the differential expression of each immune checkpoint gene (CPM) across various risk groups in TCGA data.

### 2.10 lc50 pharmaceutical analysis

The IC50 value, also known as the 50% inhibitory concentration, is the drug concentration at which apoptotic cells comprise half of the total cell count, indicative of cellular tolerance to drugs; lower IC50 values denote increased cellular sensitivity to drugs. IC50 drug analysis was conducted based on the expression levels of cancer samples, utilizing the cgp2014 database and the R package “pRRophetic” (30). Samples were categorized into high and low-risk groups based on Lasso-derived risk values, with subsequent calculation of IC50 score differences between these groups.

### 2.11 Fluorescence *in situ* hybridization

FISH was performed to validate the expression of the target genes. Briefly, tissue sections were dewaxed by sequential immersion in xylene (15 minutes each, twice) and dehydrated through a graded ethanol series, followed by rinsing in distilled water. For antigen retrieval, sections were digested with 10 μg/mL protease K in PBST at 37°C for 20 minutes and washed three times with PBS (5 minutes each). Pre-hybridization was carried out using salmon sperm DNA diluted in hybridization buffer (1:100) at 37°C for 60 minutes. Subsequently, hybridization was performed by incubating the sections with the probe-containing hybridization solution overnight at 42°C. Post-hybridization, sections were washed sequentially with 2×SSC, 1×SSC, and 0.2×SSC at 37°C. For signal detection, HRP-conjugated streptavidin was applied and incubated at 37°C for 30 minutes, followed by PBST washes. Signal amplification was achieved using TSA reagent (20 minutes incubation), and nuclei were counterstained with DAPI for 10 minutes at room temperature in the dark. Finally, sections were mounted with an anti-fade mounting medium and imaged using an inverted fluorescence microscope (Nikon, Japan).

### 2.12 Cell culture and transfection

Human melanoma cell lines (A375, SK-MEL-28) were generously provided by the First Affiliated Hospital of China Medical University. A375 and SK-MEL-28 human melanoma cells were cultured respectively in Minimum Essential medium Eagle (MEM, Procell Life Science&Technology Co., Ltd., Wuhan, China) and Dulbecco’s modified Eagle’s medium (DMEM, Procell Life Science&Technology Co., Ltd., Wuhan, China) supplemented with 10% fetal bovine serum (VivaCell, Shanghai, China) and 1% penicillin/streptomycin (Procell, China). All cells were grown at 37°C in a cell incubator with 5% CO_2_. *LINC02132* and *COPDA1* were constructed by Gene Pharma (Shanghai, China) for *LINC02132* and *COPDA1* overexpression. Briefly, we cultured cells using six-well plates the day before transfection, once the cell confluence was reached 90%. A375 and SK-MEL-28 human melanoma cells were infected with *LINC02132* and *COPDA1* or empty vector by lipo2000 (ThermoFisher) according to the ThermoFisher. After 36h transfection, the cells were conducted for further research.

### 2.13 RNA extraction and qRT-PCR assays

Total RNA was extracted from transfected cells using SteadyPure Quick RNA Extraction kit (Accurate Biotechnology, China) which according to the manufacturer’s instructions. RNA quality was analyzed by agarose gel, Nanodrop (ND-2000; ThermoFisher). Next, under the help of reverse transcription kits (NovoProtein, NJ, USA), we obtained cDNA. PCR of *COPDA1* and *LINC02132* was performed using a SYBR Greenbased qPCR kit (NovoProtein, NJ, USA). qRT-PCR was conducted using a real-time qPCR instrument (). The primers were synthesized by Sangon Biotech and the sequences used in this study were as follows: *COPDA1* F: 5’-TCTCCATCCCAGCTCGCCTTTG-3’, R: 5’-TGCCGCATCCCGTCAGGTTC-3’; *LINC02312* F: 5’-TCGCTTCTCCAGCCTTTA-3’, R: 5’-TTTGCCCTGAGCAGTTCC-3’. Relative expression levels of LncRNA were normalized to the GAPDH. We recorded the CT values of each well and calculated by using the 2^­ΔΔCt^ method.

### 2.14 Edu assays

Cells were implanted into 12-well plates and transfection 36h. The next day, edu was diluted with culture medium, and an appropriate volume of edu was added to the each well for 2h at 37°C. Subsequently, the cells were fixed with 4% paraformaldehyde for 20min and permeated by 0.3% triton. Each well was added 200μL of edu click reaction solution, and cell nuclei were labeled by Hoechst 33342 (Beyotime, Shanghai, China). Lastly, the treated cells were observed by laser scanning microscope.

### 2.15 Wound healing assays

Cells were cultured on 6-well plates the night before and transfected the next day. After 36h, we used a small pipette tip to scrape the cell monolayer. Cells were then washed with PBS 2-3 times, we added serum-free medium and cultured the cells further. Wound closure was imaged using a microscope at 0 and 24h. The migration of cells was determined based on the wound area at 0h and 24h.

### 2.16 Transwell migration and invasion assays

The cells were trypsinized into single cells and suspended in a serum-free medium. 3 x 10^5^ cells were inoculated in the upper chamber with serum-free medium. Then, 500μL of DMEM or MEM with 10%FBS was added in the lower chamber, and the cells were incubated for 48h. Then, the upper chamber was washed with PBS, and the cells in the upper chamber were wiped off. Next, we fixed the lower chamber with 4% paraformaldehyde for 30 minutes and stained with crystal violet solution for 20 minutes. The chambers were washed with PBS, and observed by microscope.

The protocol for invasion assays was basically the same as that for migration assays with the addition of Matrigel (Servicebio, China) to the lower chamber. The cell numbers were counted with Image J.

### 2.17 Flow cytometry for apoptosis detection

The rate of apoptosis in cells was evaluated using an annexin V-FITC/propidium iodide (PI) apoptosis detection kit (Beyotime, Shanghai, China). The cells were digested with EDTA-free pancreatic enzymes, centrifuged, and washed twice with PBS. Then, cells were re-suspended with binding buffer and mixed with annexin V-FITC and PI. Mixed cells were incubated on ice for 20min away from light, and then analyzed using flow cytometry.

### 2.18 Detection of intracellular ROS

To observe whether *LINC02132* and *COPDA1* would increase intracellular ROS accumulation, we used a ROS detection kit (Beyotime, Shanghai, China). The cells were cultured on 12-well plates and then incubated with 10μmol/L DCFH-DA at 37°C for 20min according to the manufacturer’s instructions. The plates were washed with serum-free medium and then observed by laser scanning microscope.

### 2.19 Statistical analysis

We use R software (version 3.5.3) to analyze the data in our study. All data were analyzed using GraphPad Prism version 9.0 software. The results are presented as the mean ± standard deviation (SD). An unpaired two-tailed Student’s t-test was utilized for the comparison of two groups, whereas a one-way ANOVA accompanied by Tukey’s *post-hoc* test was employed for the evaluation among multiple groups. P<0.05 was considered to indicate statistical significance (ns, P≥0.05, ^*^p<0.05, ^**^p<0.01, ^***^p<0.001, ^****^p<0.0001).

## 3 Results

### 3.1 Identification of DE-OSlncRNAs

**Figure 1:**
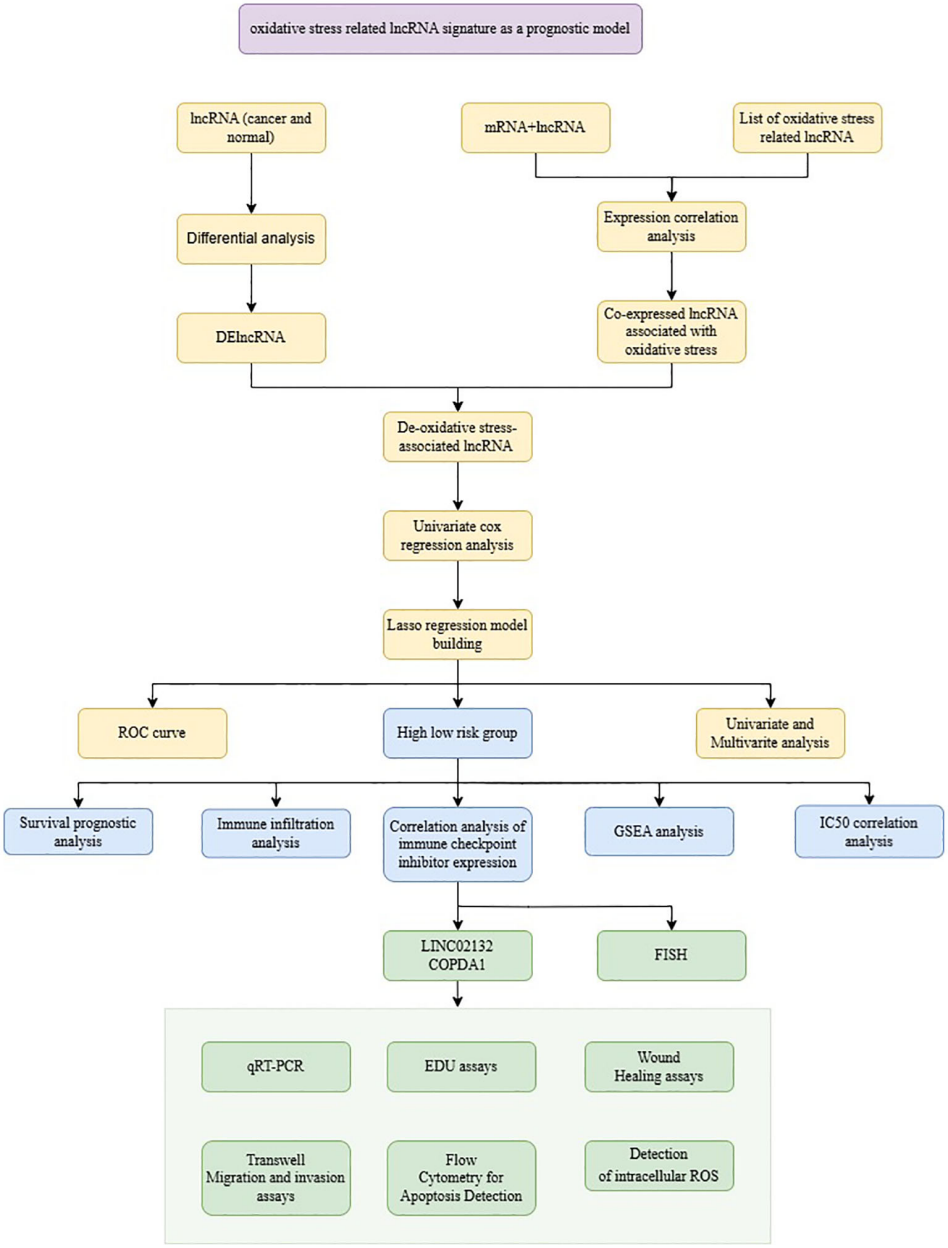
The flowchart of this study.

**Figure 2:**
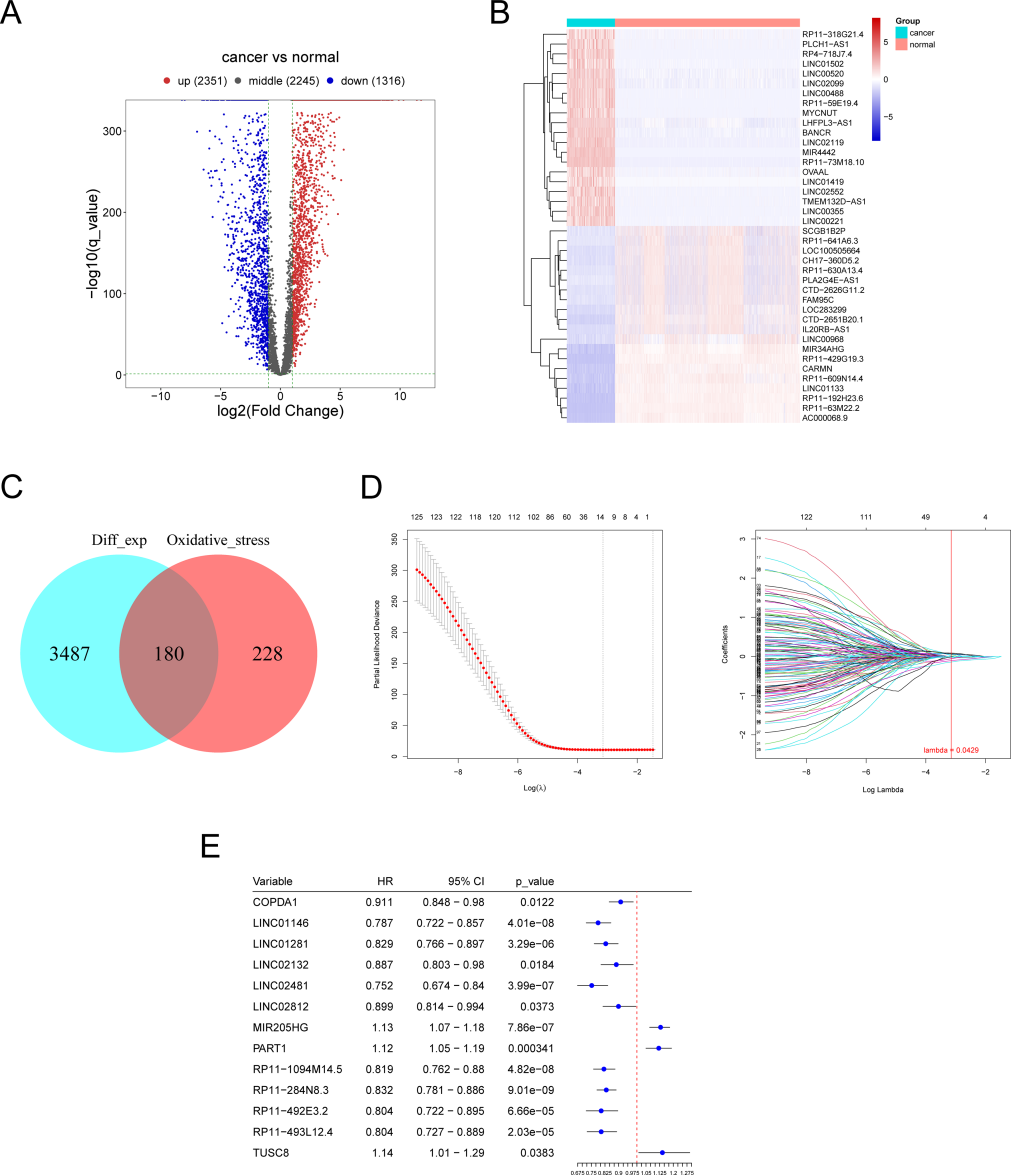
Identification of differentially expressed oxidative stress-related lncRNAs (DE-OSlncRNAs). **(A)** Volcano plot illustrating the differential expression of genes. **(B)** Clustering heatmap of the top 20 downregulated genes. **(C)** Venn diagram showing DE-OSlncRNAs. **(C)** Lasso regression of prognostic melanoma-related lncRNA model. **(D)** Lasso regression of prognostic melanoma-related lncRNA model. **(E)** Results of univariate Cox regression analysis for marker genes.

The flow chart of the whole study is shown in **Figure 1**. Differential expression analysis was conducted on the RNA sequencing data from skin cutaneous melanoma and the GTEx database using the edgeR package. Additionally, genes exhibiting differential expression and oxidative stress-related lncRNAs were identified using specified parameters. Upon comparing cancer samples to normal samples, it was found that 2351 genes were upregulated and 1316 genes were downregulated. The results are depicted in **Figure 2A**. In the subsequent analysis, we constructed a more intuitive clustering heatmap to represent the top 20 upregulated and downregulated genes (**Figure 2B**). Subsequently, lncRNAs exhibiting differential expression and high correlation with oxidative stress genes (correlation index >0.6) were selected (**Figure 2C**). This resulted in 180 intersecting genes. Univariate Cox regression analysis was then performed on these lncRNAs associated with oxidative stress in the TCGA dataset, based on both expression values and clinical information. This analysis identified 125 differentially expressed lncRNAs with a P-value < 0.05 (**Figure 2D**).

Lasso candidate lncRNAs were selected based on the prior screening, and a Cox regression model was constructed using glmnet, with TCGA samples divided into training and testing groups in a 1:1 ratio. The regression model was built on the training group and then validated using the test group and the entire sample set. As depicted in **Figure 2E**, thirteen lncRNAs were identified as significant prognostic factors. The risk score formula was as follows: (0.049 × *COPDA1* expression) + (-0.015 × *LINC01146* expression) + (-0.058 × *LINC01281* expression) + (-0.018 ×*LINC02132* expression) + (-0.054 × *LINC02481* expression) + (0.069 × *LINC02812* expression) + (0.07 × *MIR205HG* expression) + (0.056 × *PART1* expression) + (-0.018 × *RP11-1094M14.5* expression) + (-0.141 ×*RP11-284N8.3* expression) + (-0.1 × *RP11-492E3.2* expression) + (-0.009 × *RP11-493L12.4* expression) + (0.088 × *TUSC8* expression). We performed differential expression analysis of the thirteen lncRNAs between cancerous and normal tissues and assessed their prognostic value by analyzing survival curves for high and low expression levels of each gene, as shown in **Supplementary Figure S1**. Genes with nonzero regression coefficients were selected as marker genes from the LASSO regression analysis, followed by univariate Cox regression analysis of these marker genes to identify significant genes. Furthermore, the risk values for each sample were predicted based on the expression values of the genes identified in the regression model.

### 3.2 A lncRNA prognostic model related to oxidative stress was constructed and validated

**Figure 3:**
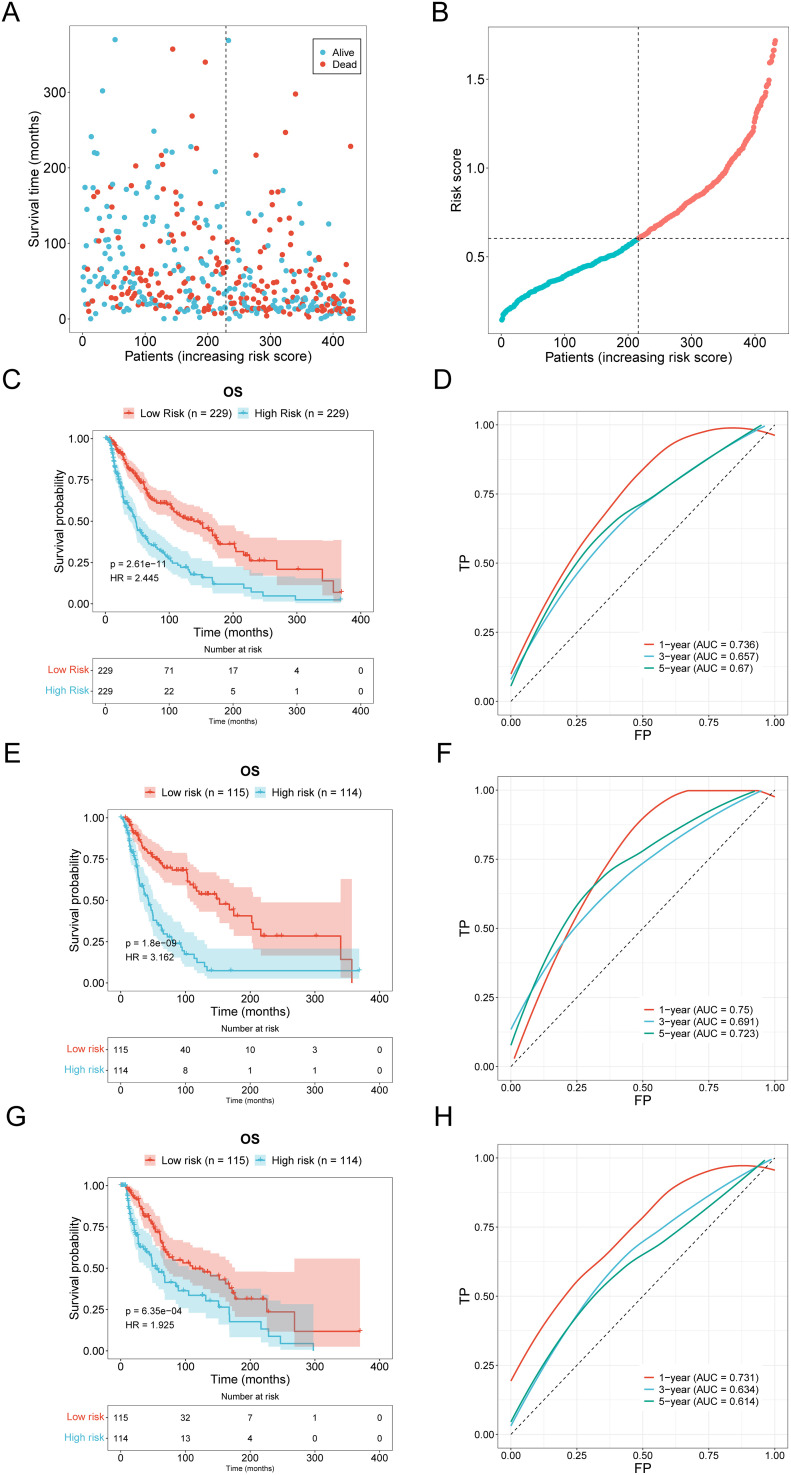
Validation of prognostic models. **(A, B)** Distribution and scatter plot of the relationship between risk score and survival time in the high-low risk group of SKCM patients. **(C, D)** The distribution and scatter plots of SKCM patients show the relationship between risk score and survival time **(E, F)** Kaplan-Meier survival curve and ROC curve of the training group. **(G, H)** Kaplan-Meier survival curve and ROC curve of the testing group.

A novel risk prediction model for evaluating the prognosis of SKCM (Skin Cutaneous Melanoma) patients. Samples were classified into high-risk and low-risk groups based on the median risk values, followed by survival analyses of the entire sample, training group, and testing group set to evaluate the model’s effectiveness. Our scatter plot results indicate that high-risk scores are inversely correlated with the survival time of SKCM (Skin Cutaneous Melanoma) patients (**Figures 3A, B**). Additionally, Kaplan-Meier survival curve analysis demonstrates that the overall survival (OS) rate of patients with high-risk scores is significantly lower than that of patients with low-risk scores and the ROC curve also showed consistent results (**Figures 3C, D**). The area under the ROC curve (AUC) was used to validate the regression model. **Figures 3F, H** demonstrates that low-risk samples had a higher survival probability than high-risk samples, with AUC values indicative of survival over 1, 3, and 5 years. The Kaplan-Meier survival curves demonstrate that the risk score based on oxidative stress-related prognostic lncRNAs are negatively correlated with survival time in both the training and testing cohorts (**Figures 3E, G**).

**Figure 4:**
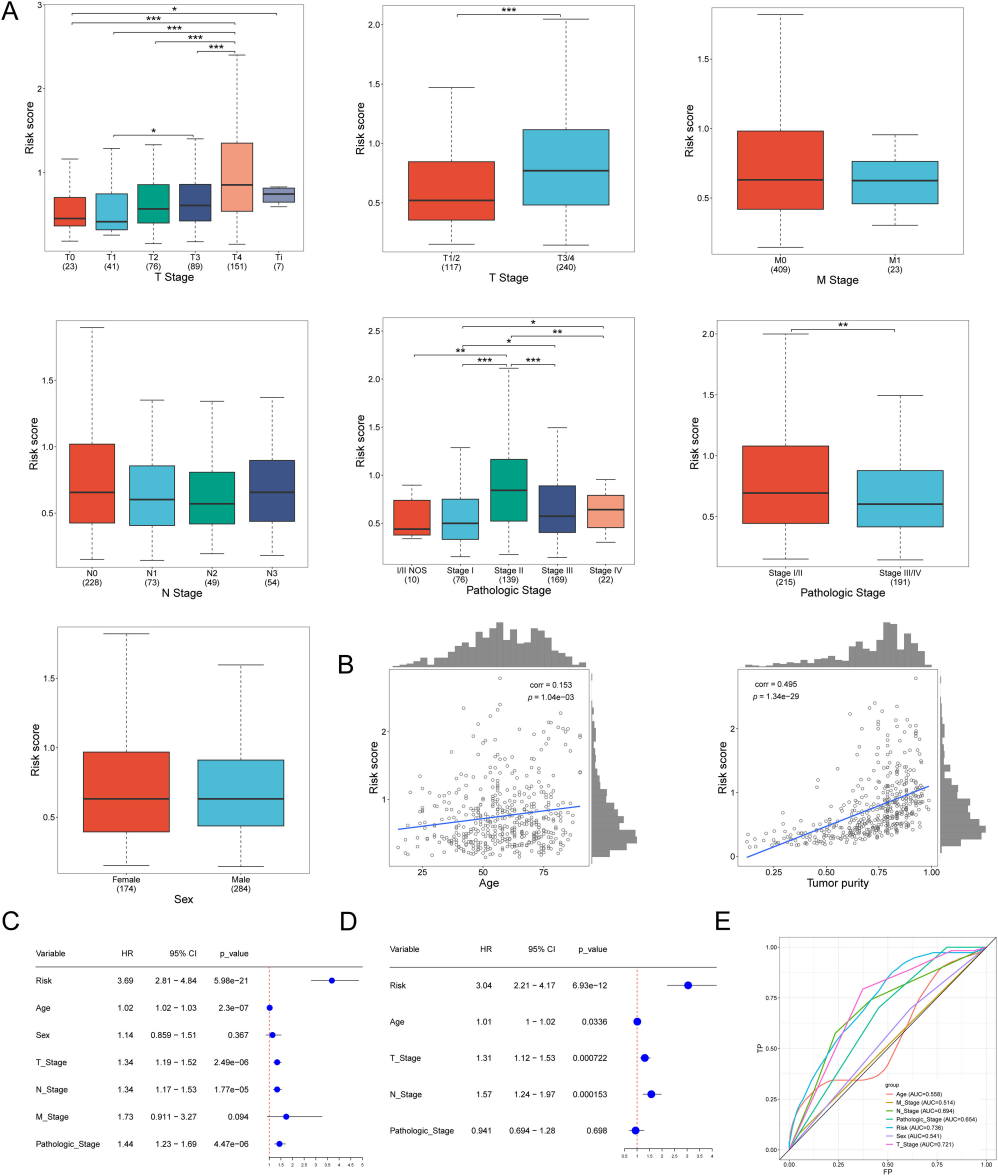
The correlation of pathological stage, age, and risk score. **(A)** Risk Score distribution across tumor stages, pathologic and sex characteristics. **(B)** Correlation graph between age and risk score. Correlation graph between tumor purity and risk score. **(C)** Univariate Cox regression revealed differences in the T, N,pathologic, and risk scores of lncRNA associated with oxidative stress as prognostic indicators of SKCM. **(D)** Multivariate Cox regression revealed differences in the T, N, and risk scores of lncRNA associated with oxidative stress as prognostic indicators of SKCM. **(E)** The ROC curve of the age, T Stage, N Stage, M Stage, Pathologic Stage, Risk, and Sex. *p<0.05, **p<0.01, ***p<0.001.

Samples were categorized into various groups based on pathological stage and sex. The risk scores across different stages were analyzed. Additionally, the risk scores for females and males were analyzed, with the results presented in **Figure 4A**. The differential conditions of each clinical group were calculated, along with the Pearson correlation between clinical data and risk values, to assess the clinical applicability of the constructed model. Correlation analysis revealed that age and tumor purity were positively correlated with risk scores, as shown in **Figure 4B**. However, other factors, such as sex and pathological stage, did not exhibit a significant correlation with risk scores. This suggests that factors like age and tumor purity are adequately represented in the constructed model.

Univariate and multivariate regression analyses can further validate the reliability and accuracy of the model. Based on the different weights of these genes, a comprehensive risk score was calculated. Univariate regression analysis showed the risk (HR = 3.69, P < 0.001), T (HR = 1.34, P < 0.001), N (HR = 1.34, P < 0.001), and Pathologic (HR = 1.44, P < 0.001). Multivariate regression analysis showed that Risk (HR=3.04, P<0.001), T (HR=1.31, P<0.001), and N (HR=1.57, P<0.001). These results (**Figures 4C, D**) demonstrate the accuracy and scientific validity of the prognostic model we constructed. We calculated the ROC results for each clinical parameter and their corresponding risk values over one year. The area under the ROC curve for Risk was 0.736, for T Stage was 0.721, for N Stage was 0.694, and for Pathologic Stage was 0.694. These results indicate that the model is highly valuable in predicting skin melanoma (**Figure 4E**).

### 3.3 Immune infiltration、immune checkpoint、ic50 pharmaceutical analysis

**Figure 5:**
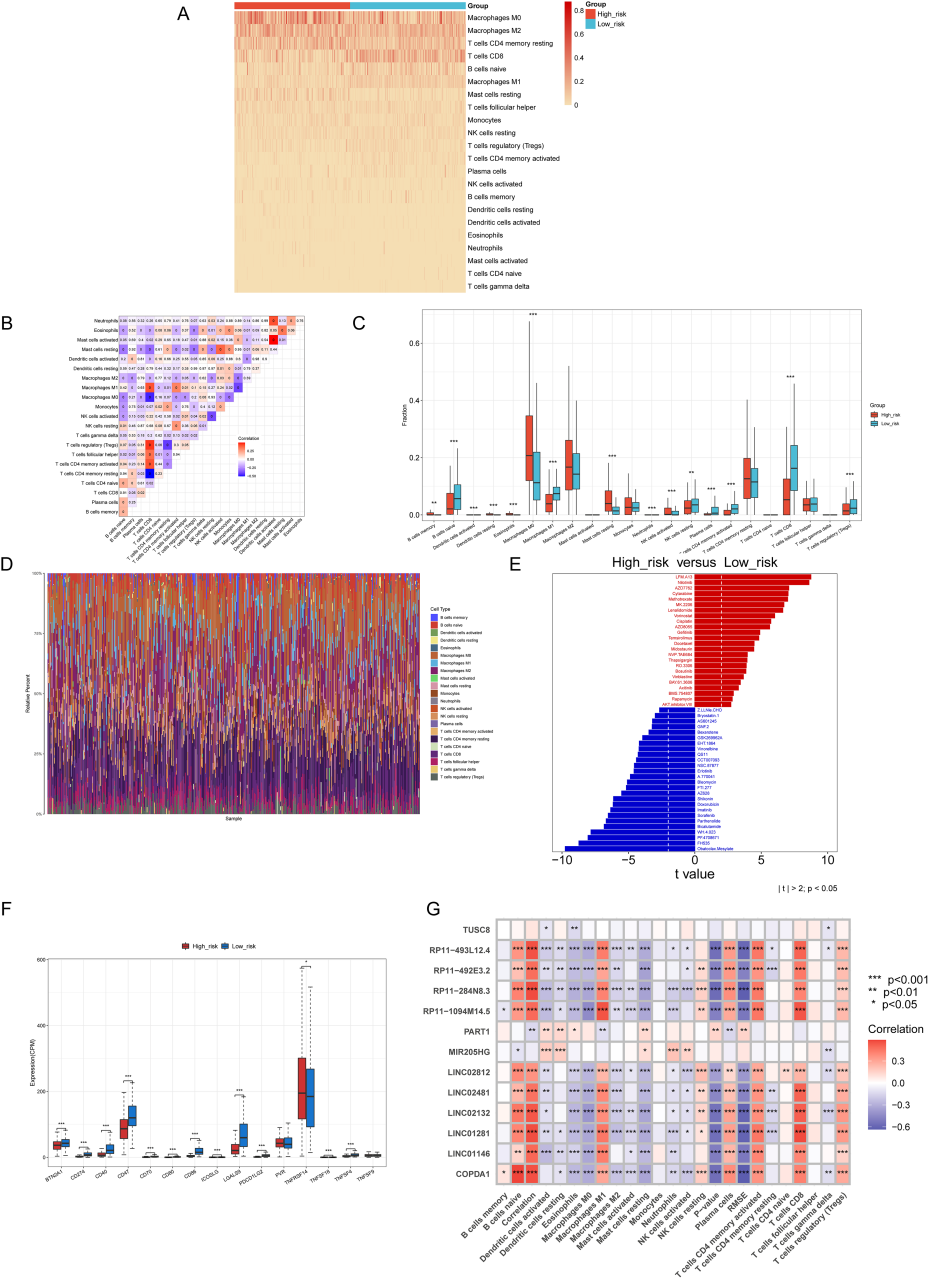
The results of Immune Infiltration、Immune Checkpoint、ic50 Pharmaceutical Analysis. **(A)** A heat map of immune infiltration scores for each sample corresponding to each cell. **(B)** A correlation heatmap detailing each immune cell type. **(C)** The distribution of 22 immune cell types in patients from the low-risk and high-risk groups, was analyzed by the CIBERSORT algorithm. **(D)** The bar diagram of immune cells illustrates the proportional distribution of cells in each sample. **(E)** Analysis of drug sensitivity differences between high- and low-risk Groups. **(F)** Differential analysis of immune checkpoints between the high-risk and low-risk groups. **(G)** Correlations between thirteen genes and immune cell types. *p<0.05, **p<0.01, ***p<0.001.

We employed CIBERSORT to determine immune infiltration scores, based on gene expression profiles of skin cutaneous melanoma (SKCM) samples from the TCGA. The results depicted in **Figures 5A–D**, revealed a higher proportion of Macrophages M0, M2, activated CD4 memory T cells, CD8 T cells, naive B cells, and Macrophages M1. Subsequently, samples were classified into high-risk and low-risk groups based on their lasso regression risk values. The differential cellular composition and immune Checkpoint of these groups were analyzed using the Wilcoxon test. The results suggested that TNFRSF14 and CD47 may be potential targets for immunotherapy (**Figure 5F**).

Next, we performed an IC50 pharmaceutical analysis based on the expression profiles of cancer samples in the TCGA RNA-seq and CGP2014 database We assessed the differential IC50 scores between the two groups. We assessed the drug sensitivity of melanoma patients by analyzing the IC50 values of various drugs, as determined by the pRRophetic package, across high-risk and low-risk groups. Among the drugs tested, several chemotherapeutic agents, such as Nilotinib, Sorafenib, GX15-070, and Cisplatin, have been reported in the literature for their potential therapeutic use in melanoma treatment (31–34). Finally, the results were depicted in a bar diagram (**Figure 5E**). The IC50 scores of LFM.A13, Nilotinib, and AZD7762 were lower in the high-risk group, while those of Obatoclax. Mesylate and FH535 were lower in the low-risk group. This enables the use of targeted drug therapies based on the specific characteristics of cutaneous skin melanoma. Furthermore, the correlation between the thirteen selected genes and the immune cell infiltration levels obtained from CIBERSORT is shown in **Figure 5G**.

### 3.4 Gene set enrichment analysis

**Figure 6:**
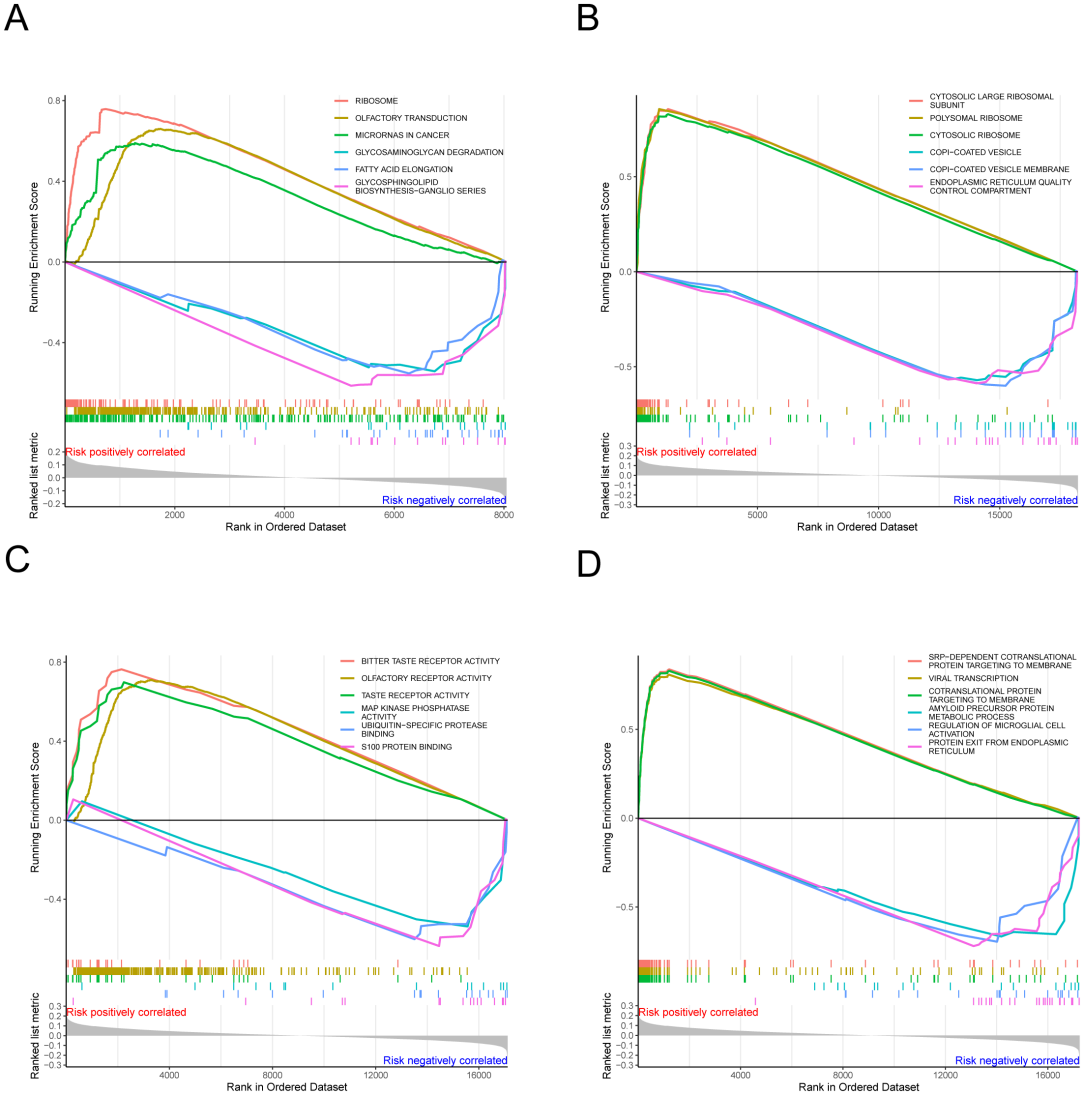
The results of gene set enrichment analysis. **(A–D)** Functional enrichment analysis, encompassing biological processes, cellular components, and molecular functions.

In both the high-risk and low-risk groups, gene set enrichment analysis (GSEA) offers significant insights into the prognosis and treatment of melanoma. Utilizing the aforementioned risk values, alongside gene expression data from each cancer sample and databases such as GO and KEGG, GSEA_v4 was employed for gene set enrichment analysis. GO enrichment analysis includes categories such as biological processes, cellular components, and molecular functions. **Figure 6D** demonstrates that SRP-dependent cotranslational protein targeting to membrane and viral transcription are positively correlated with cutaneous skin melanoma in high-risk groups. Conversely, processes like amyloid precursor protein metabolic process, regulation of microglial cell activation, and protein exit from the endoplasmic reticulum are negatively correlated in the biological process category. Furthermore, components such as cytosolic large ribosomal subunits, polysomal ribosomes, and cytosolic ribosomes were positively correlated with cutaneous skin melanoma in the cellular component category, while COPI-coated vesicles, COPI-coated vesicle membranes, and endoplasmic reticulum quality control compartments showed a negative correlation (**Figure 6B**). In the molecular function category, activities such as bitter taste receptor, olfactory receptor, and taste receptor were positively correlated with cutaneous skin melanoma, whereas MAP kinase phosphatase binding and S100 protein binding exhibited negative correlations (**Figure 6C**). The KEGG pathway enrichment analysis (**Figure 6A**) indicated that pathways such as ribosome, olfactory transduction, and microRNAs in cancer were positively correlated with cutaneous skin melanoma. These results can be leveraged to enhance melanoma prognosis.

### 3.5 FISH method to validate

**Figure 7:**
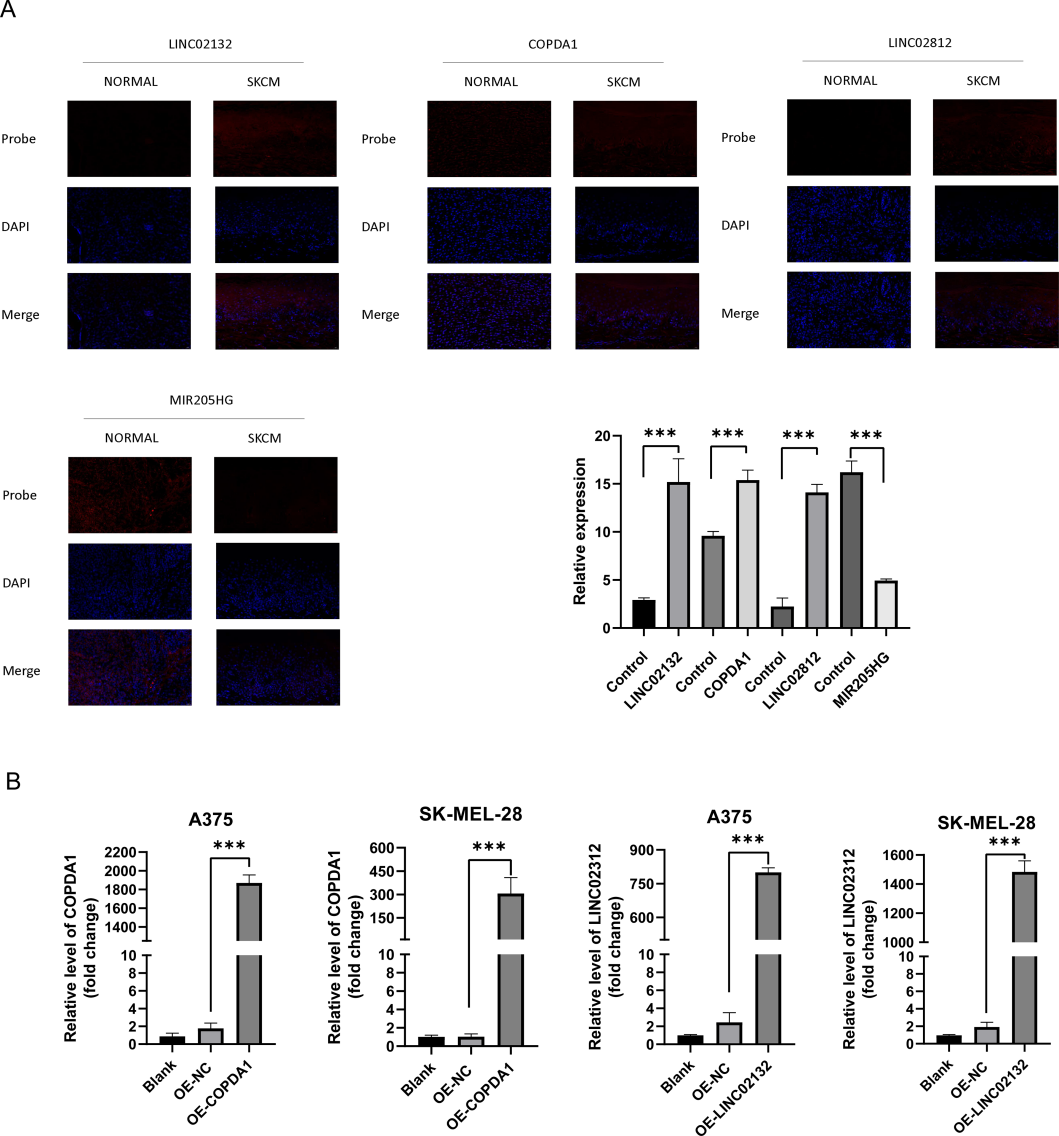
Gene expression of *COPDA1* and *LINC02132*. **(A)** Applications of Fluorescence *in situ* Hybridization in the cancer and normal groups. The figures depict the staining patterns of the four probes: *LINC02132, COPDA1*, *LINC02812*, and *MIR205HG*. DAPI staining highlights the nuclei, shown in blue, and the four probes are depicted in red, enabling a clear distinction between the two groups. In the SKCM group, *LINC02132*, *COPDA1*, and *LINC02812* showed increased expression compared with the normal group, while *MIR205HG* expression was significantly reduced. We collected tissue sections from 11 melanoma patients, totaling 44 slides, and 4 normal skin samples, totaling 12 slides. Melanoma group, n=11; normal group, n=4. **(B)** The expression *COPDA1, LINC02132* in the blank, negative control (NC), OE-COPDA1 and OE-LINC02132 groups was detected using qRT-PCR. Data are as shown as mean ± SD; ***P < 0.001.

Previous studies have demonstrated the significant reference value of FISH (Fluorescence *in situ* Hybridization) in diagnosing melanoma (35). FISH employs a fluorescent staining method to intuitively observe chromosome numbers, thereby assessing tumor cell proliferation and identifying risk genes (36). We stained a total of 48 sections, comprising 44 melanoma sections and four normal skin sections. From these, we selected the appropriate field of view separately, and analysis of fluorescence areas allowed for inference of risk and protective factors. The results were consistent with previous findings (**Figure 7A**). Compared to the melanoma cell group, expression of *COPDA1*, *LINC02132*, and *LINC02812* in the cytoplasm was elevated in the normal skin group. However, in the normal skin group, *MIR205HG* expression in the cytoplasm was found to be decreased.

### 3.6 Tumor suppressor effect of *LINC02132* and *COPDA1* overexpression

**Figure 8:**
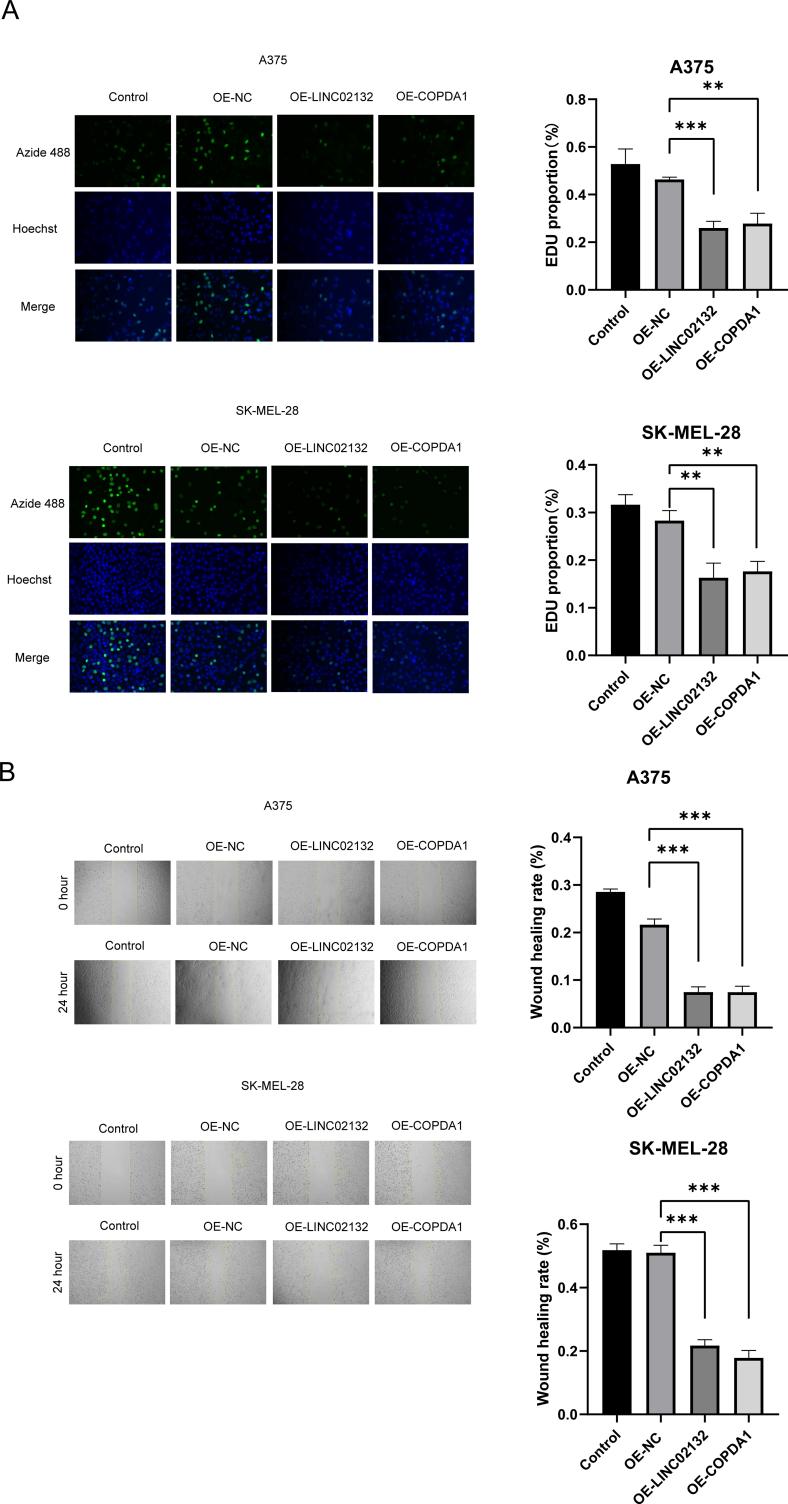
The effects of *LINC02132* and *COPDA1* on cell proliferation and migration in melanoma cell lines. **(A)** The effect of *LINC02132* and *COPDA1* transfection on A375 and SK-MEL-28 cells proliferation was detected by EDU assay. **(B)** The effect of *LINC02132* and *COPDA1* on the migratory ability of A375 and SK-MEL-28 cells was assessed by the scratch wound assay. Data are as shown as mean ± SD; **P < 0.01, ***P < 0.001.

**Figure 9:**
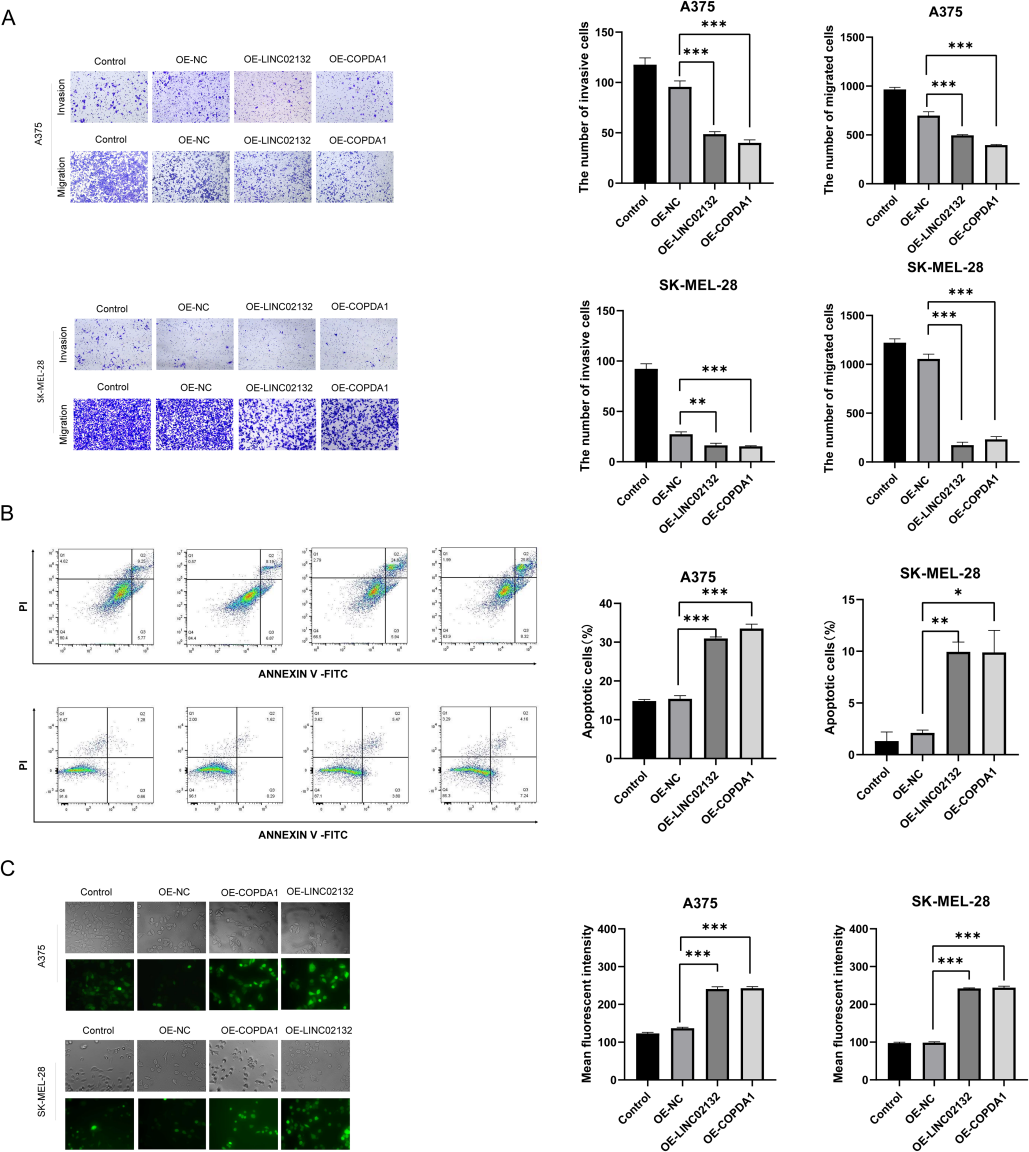
The effects of *LINC02132* and *COPDA1* on cell invasion, apoptosis, and intracellular ROS levels in melanoma cell lines. **(A)** In transwell assays without Matrigel, the over-expression of *LINC02132* and *COPDA1* inhibited the migration capacity of melanoma cells. In Matrigel invasion assays, over-expression of *LINC02132* and *COPDA1* inhibited the invasive capacity of melanoma cells **(B)** Cell apoptosis rate was tested via flow cytometry analysis. **(C)** The intracellular ROS content of melanoma cells after transfection was detected, image J was used to analyze the fluorescence density of the samples. Data are as shown as mean ± SD; *P < 0.05, **P < 0.01, ***P < 0.001.

Two melanoma cell lines, A375 and SK-MEL-28, were used for validation *in vitro*. We transfected the overexpression plasmids of both genes, and qRT-PCR detection showed that the overexpression was more than several thousand times, which means that we successfully transferred the plasmid into the cells (**Figure 7B**). The results of EDU proliferation assay showed that the cell proliferation activity increased by *LINC02132* and *COPDA1* expression was inhibited (**Figure 8A**). The above results were further confirmed by live cell reactive oxygen species (ROS) staining, and the intracellular ROS level was significantly increased after overexpression of *LINC02132* and *COPDA1* (**Figure 9C**). In order to further verify the effect of *LINC02132* and *COPDA1* genes on the migration and invasion of melanoma cells, we performed scratch assay and transwell invasion and invasion assay, and the results showed that *LINC02132* and *COPDA1* could reduce the migration and invasion of melanoma cells (**Figures 8B**, **9A**).

### 3.7 *LINC02132* and *COPDA1* regulate melanoma cells apoptosis progression

To further investigate the effect of *LINC02132* and *COPDA1* on the apoptosis of melanoma cells, we transfected the overexpression plasmid through flow cytometry. We observed that melanoma cells overexpressing *LINC02132* and *COPDA1* had an increased number of apoptotic cells compared with the control group (**Figure 9B**). These results confirmed that *LINC02132* and *COPDA1* could accelerate the apoptosis of melanoma cells.

## 4 Discussion

Cutaneous skin melanoma (SKCM) is characterized by a low cure rate, high mortality rate, and limited predictability (37). Current therapy methods are not highly effective, but there has been significant progress in recent years, including advancements in immunotherapy, targeted therapy, radiation therapy, and surgical operations (38). There is evidence that immune checkpoint inhibitors can effectively improve the prognosis of melanoma patients, and it is important to note the drivers of response related to cell composition and the microenvironment (39). Intracellular ROS can be categorized into two main states: redox homeostasis and oxidative stress. Under normal conditions, ROS maintains cellular physiological functions, representing redox homeostasis. In contrast, elevated levels of ROS lead to oxidative stress (40). Oxidative stress is closely linked to the initiation and progression of melanoma. The accumulation of reactive oxygen species (ROS) and related products within cells can permeate the mitochondrial membrane, leading to DNA damage and disruption of the tumor microenvironment. These effects further influence cellular proliferation and apoptosis (41, 42). In recent years, lncRNAs have garnered significant attention, as they constitute the majority of the genome compared to miRNAs and mRNAs. Studies have shown that the number of lncRNAs may exceed that of protein-coding transcripts, and their dysregulation and mutations are associated with numerous diseases, particularly cancer. Furthermore, lncRNAs are involved in various cellular functions, such as cell differentiation, apoptosis, and autophagy (43–45). Due to their tissue-specific and cell type-specific expression, lncRNAs can serve as specific biomarkers for disease subtypes. Their regulatory mechanisms are relatively complex, as they can form intricate regulatory networks with miRNAs and mRNAs, thereby providing a more comprehensive framework for understanding gene regulation (46). Previous studies have revealed that an oxidative stress-related lncRNA signature is closely associated with melanoma progression (47). Additionally, some antioxidant enzymes can mitigate tumor cell proliferation by regulating ROS levels and T-cell activation ( 48). This holds significant value for the prognosis of melanoma patients (49). Our study focuses on utilizing the oxidative stress-related lncRNA signature to develop a prognostic model and explore new predictive signatures. Additionally, our study offers novel insights into prolonging the lifespan of melanoma patients.

Initially, we selected 180 metabolism-related differentially expressed genes (DE-OSlncRNAs) and utilized univariate Cox regression analysis to identify prognostically relevant DE-OSlncRNAs. We constructed a prognostic model and employed a ROC curve to verify its predictive capability. The results indicated that, across training and testing groups, as well as all samples, the low-risk group exhibited a higher overall survival rate than the high-risk group. Subsequently, we identified marker genes according to the prognostic model, categorizing them into those that inhibit and those that promote tumor proliferation. A key advantage of our study is the use of FISH to test our representative marker genes, including *COPDA1*, *LINC02132*, *LINC02812*, and *MIR205HG*, thereby enhancing the experiment’s reliability and accuracy.

*COPDA1* has been implicated in the occurrence and development of COPD (50). *LINC02132* and *LINC02812*, both Long Intergenic Non-Protein Coding RNAs (lncRNAs), are RNA genes affiliated with the lncRNA class. Dong et al. reported variations in *MIR205HG* expression, either increasing or decreasing across different tumors (51). In our experiments, *MIR205HG* emerged as a risk factor for SKCM, with increased expression observed in the cytoplasm. These four marker genes enhance the detection rate of SKCM and overall survival (OS). The abnormal expression of these lncRNAs is reasonably suspected to be closely related to the occurrence and prognosis of melanoma. Furthermore, the samples were categorized into low-risk and high-risk groups. We also considered the association between pathological factors and risk values, calculating the Pearson correlation between clinical data and risk values. The results indicated that the pathologic stage can significantly influence the degree of risk. Additionally, we verified that both age and tumor purity are highly positively correlated with risk.

Moreover, we conducted immunoinfiltration analysis, immune checkpoint difference analysis, and drug sensitivity analysis for different groups, believing these indicators to be crucial for predicting and prognosticating SKCM. A greater abundance of immune cell components, particularly macrophages, was observed in the high-risk group. In the immune checkpoint difference analysis, TNFRSF14 (52) levels were elevated in high-risk groups, while CD47 and LGAL59 also played indicative roles in low-risk groups. Our study indicates that patients in different risk groups respond to different drugs, suggesting tailored treatment approaches for SKCM based on patient risk profiles. Finally, we observed numerous pathways positively enriched in the KEGG database, such as the ribosome (53), olfactory transduction, and microRNA in cancer (54), potentially associated with oxidative stress-related lncRNA. The GO/KEGG analysis results for SKCM suggest that the prognosis of cutaneous skin melanoma is correlated with certain biological processes, including SRP-dependent cotranslational protein targeting to the membrane, viral transcription, and cotranslational protein targeting to the membrane. Research has demonstrated that membrane proteins are positively associated with melanoma (55), presenting potential therapeutic targets and marker genes.

Finally, we conducted an IC50 pharmaceutical analysis between the high-risk and low-risk groups. Previous studies have shown that melanoma cell proliferation is associated with the AKT, PI3K, mTOR pathways at the protein level (37). These results also highlight the potential efficacy of chemotherapy drugs such as AKT inhibitor VIII (56), Rapamycin (57) BMS-754807, Z-LLNle-CHO, Bryostatin-1, and AS601245. Costunolide, an AKT inhibitor, has been shown to inhibit melanoma cell proliferation (58), and BMS-754807, an IGF1R/IR inhibitor, can also suppress tumor cell growth (59). Currently, there is a lack of definitive studies confirming that Z-LLNle-CHO inhibits cancer cell proliferation and improves survival rates and prognosis in high-risk patients. Meierjohann, S. found that oxidative stress and certain injuries can lead to melanocytic multinucleation, increasing melanoma cell resistance to drugs (60). Z-LLNle-CHO may improve treatment outcomes for melanoma patients and is a potential focus for future research.

In addition, our study has the following limitations. While we utilized public databases and validated our findings through cell and tissue slice experiments, clinical samples were relatively limited. Melanoma has many subtypes in clinical practice, but detailed clinical information is often lacking due to the limitations of public databases. We can also investigate the specific regulatory mechanisms of *LINC02132* and *COPDA1* on melanoma cell apoptosis in future experiments. We hope that clinical data can be further improved allowing us to correlate lncRNA expression levels with patient treatment outcomes and make our research more rigorous. Furthermore, if conditions permit in the future, the remaining genes identified through our bioinformatics screening should also be experimentally validated. Moreover, we are able to conduct predictive analysis regarding the specific mechanisms underlying lncRNA-mediated ceRNA networks. In conclusion, we utilized multiple databases to establish an oxidative stress-related lncRNA model. We then validated the verifiable genes, *LINC02132* and *COPDA1*, in two melanoma cell lines and clinical tissues. The results demonstrated that both *LINC02132* and *COPDA1* influence the proliferation, migration, and invasion of melanoma cells. This has provided us with a more comprehensive understanding of the potentially predictive genes associated with melanoma. Simultaneously, our study aids in the identification of new antioxidant medications and assists in formulating personalized therapeutic approaches.

## Data Availability

The datasets presented in this study can be found in online repositories. The names of the repository/repositories and accession number(s) can be found in the article/**Supplementary Material**.

## Supplementary material

Supplementary Figure 1
**(A)** The expression levels of the thirteen differentially expressed genes in normal skin versus melanoma tissues. **(B)** Kaplan-Meier survival curves for the thirteen differentially expressed genes between the high-risk and low-risk groups. *P < 0.05, **P < 0.01, ***P < 0.001.
