# Supplementary figures and images for: Inhibiting melanoma tumor growth: the role of oxidative stress-associated LINC02132 and COPDA1 long non-coding RNAs
Source: Front Immunol. 2025 Feb 28;16:1558292. doi: 10.3389/fimmu.2025.1558292 (PMC11906686; doi:10.3389/fimmu.2025.1558292)

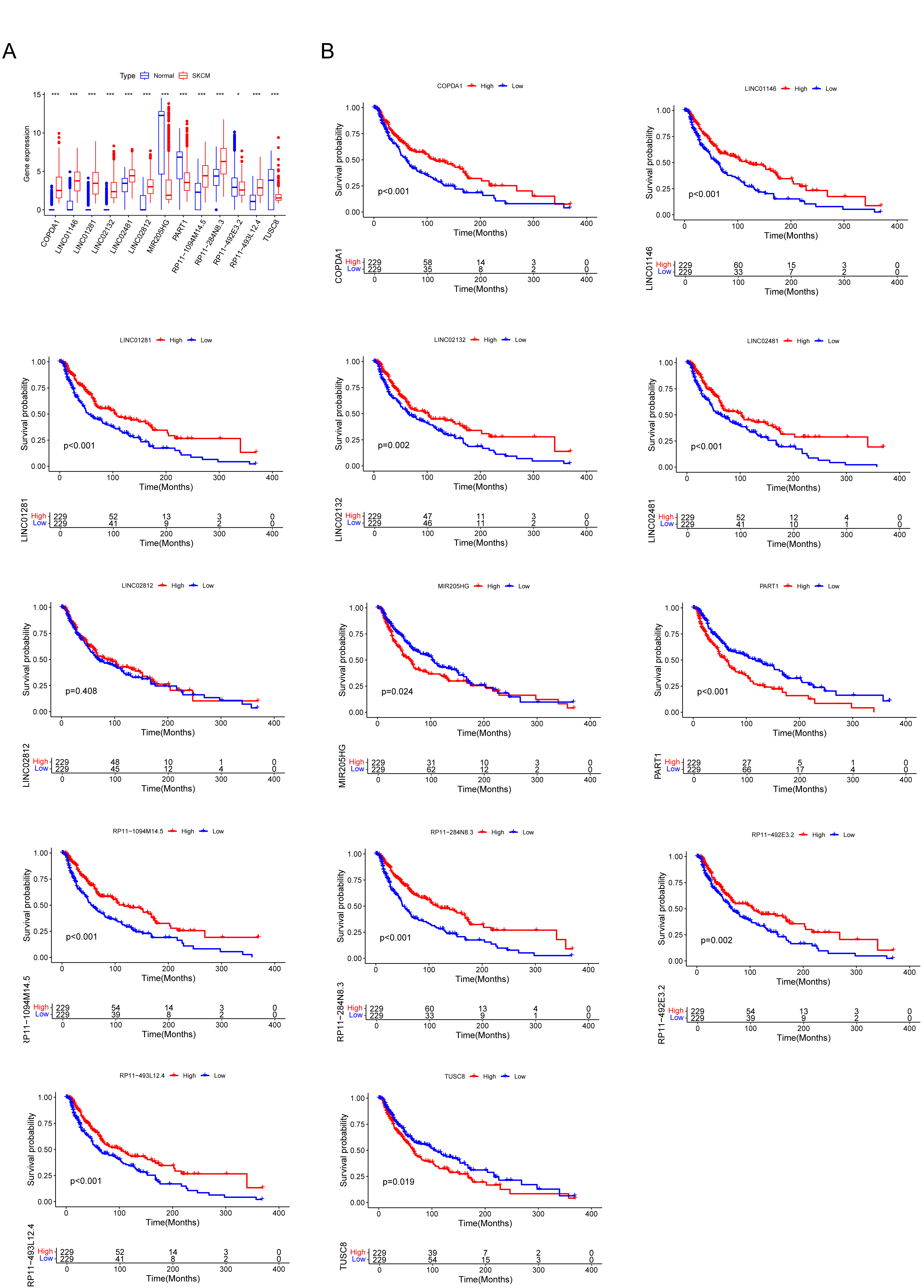

Supplement: Supplementary Figure 1 — (A) The expression levels of the thirteen differentially expressed genes in normal skin versus melanoma tissues. (B) Kaplan-Meier survival curves for the thirteen differentially expressed genes between the high-risk and low-risk groups. *P < 0.05, **P < 0.01, ***P < 0.001. [file Image1.tif]
